# A novel approach to visualize clinical benefit of therapies for chronic graft versus host disease (cGvHD): the probability of being in response (PBR) applied to the REACH3 study

**DOI:** 10.1038/s41409-023-02128-8

**Published:** 2023-10-28

**Authors:** Norbert Hollaender, Ekkehard Glimm, Jennifer Gauvin, Tommaso Stefanelli, Robert Zeiser

**Affiliations:** 1 Novartis Pharma AG, Basel, Switzerland; 2 Novartis Pharmaceuticals Corporation, East Hanover, NJ USA; 3 Department of Medicine I, Faculty of Medicine, Medical Centre, University of Freiburg, Freiburg, Germany

**Keywords:** Biostatistics, Haematopoietic stem cells

## Abstract

Overall response rate (ORR) is commonly used as key endpoint to assess treatment efficacy of chronic graft versus host disease (cGvHD), either as ORR at week 24 or as best overall response rate (BOR) at any time point up to week 24 or beyond. Both endpoints as well as duration of response (DOR) were previously reported for the REACH3 study, a phase 3 open-label, randomized study comparing ruxolitinib (RUX) versus best available therapy (BAT). The comparison between RUX and BAT was performed on ORR and BOR using all randomized patients, while DOR was derived for the subgroup of responders only. Here we illustrate the application of the probability of being in response (PBR), a graphical method presenting simultaneously the time to first response and subsequent failure using all randomized patients. In REACH3, PBR showed an earlier time to first response, a higher probability of being in response and a longer duration of response for RUX compared to BAT. PBR is a clinically easily interpretable measurement and can serve as a novel efficacy endpoint to assess treatments for chronic graft versus host disease.

## Introduction

Many studies investigating the treatment of graft versus host disease (GvHD) use overall response per 2014 NIH consensus criteria as the primary efficacy endpoint [1]. The overall response rate (ORR) is defined as the proportion of patients who achieve a complete response (CR) or a partial response (PR). The overall response rate (ORR) may be reported at a fixed time point or derived from the best overall response (BOR) achieved at any time point during treatment.

ORR at week 24 was used as primary endpoint in REACH3, a randomized phase 3 study comparing ruxolitinib versus best available therapy (BAT) for glucocorticoid-refractory chronic GvHD (cGvHD), and BOR at any time point up to week 24 was used as one of the secondary endpoints [2]. Both ORR at week 24 and BOR were higher for ruxolitinib than in the control group (BAT). To investigate how long the response to treatment is maintained, the duration of response (DOR) was calculated for all patients who achieved BOR = CR or PR, results were also reported in Zeiser et al. [2]. As DOR is computed for responders only, a formal statistical test to compare DOR between the two treatment arms was not performed since such a comparison would not be based on all randomized patients. Another measure of potential clinical interest is the time to the first response, which can be either calculated for responders only (e.g., all patients who achieved BOR = CR or PR) or for all randomized patients.

In this paper, we illustrate the application of the so-called probability of being in response function (PBR function), an extension of Kaplan–Meier estimation which facilitates the simultaneous graphical representation of the time to first response and subsequent failure, i.e., combining time to first response, response rates, and DOR into one easily interpretable measure using all randomized patients. PBR was introduced by Temkin et al as a non-parametric method to estimate the response probability as a function of time [3]. Begg and Larson and Ellis et al suggested estimating the PBR from parametric models [4, 5].

## Materials and methods

### REACH3 study and efficacy endpoints

REACH3 was an open-label randomized controlled study investigating the efficacy and safety of ruxolitinib versus BAT in patients 12 years or older with glucocorticoid-refractory or dependent cGvHD. Overall, 329 patients were randomized; 165 and 164 patients were assigned to the ruxolitinib and BAT arm, respectively. All patients (or their guardian) provided informed consent. Response evaluation was performed according to the 2014 NIH consensus criteria, response assessments were made regularly (e.g., every 4 weeks up to week 24) as per study protocol [1]. ORR at week 24, defined as the proportion of patients who achieved a CR or a PR 24 weeks after randomization, was used as primary endpoint. Other efficacy endpoints included the BOR defined as proportion of patients who achieved overall response (CR or PR) at any time point up to and including week 24, the DOR which was derived for the subset of patients with BOR = CR or PR only and failure-free survival (FFS), defined as time from randomization to recurrence of underlying disease, start of new systemic treatment for cGvHD, or death, whichever came first. An overview of selected efficacy endpoints is given in supplementary material S1. More details on the REACH3 study design and results can be found in Zeiser et al. [2].

### Probability of being in response function

**Fig. 1:**
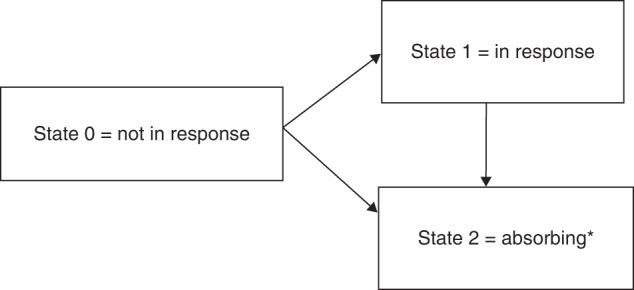
Multistate model for the response status. Illustration of multistate model for response status. Absorbing events were either death, start of new systemic cGvHD therapy, underlying malignancy relapse, not having achieved a response up to week 24 (non-responders only) or cGvHD progression (responders only). *Patient cannot achieve a response anymore after entering State 2.

**Fig. 2:**
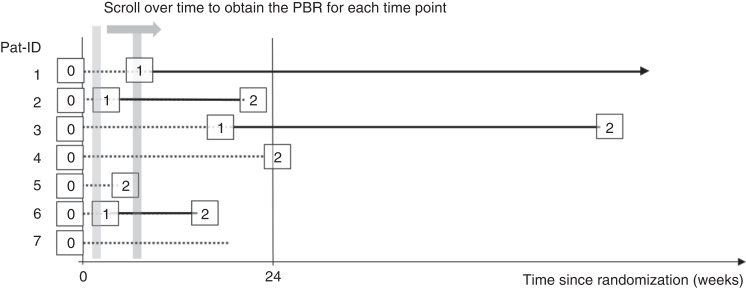
Illustration of response states over time and the concept of the PBR. Graphical illustration of response states overtime for seven hypothetical patients. 0, State 0, not in response; 1, State 1, in response; 2, State 2, absorbing; Pat-ID, patient ID.

The probability of being in response function (PBR function) suggested by Temkin can be derived from a multistate model (Fig. 1) [3]. As illustrated for seven hypothetical patients in Fig. 2, all patients are in state 0 (*not in response*) at baseline (=randomization date). A patient responding to treatment enters state 1 = *in response* at the time of the first documented response. Such patients may lose their response at a later time point and enter the *absorbing state*, state 2, (Pat-ID 2, 3, and 6) or remain *in response* at the time of the statistical analysis (Pat-ID 1), in which case they are censored in state 1. Patients who die or progress or start a new systemic cGvHD treatment or do not achieve response within 24 weeks switch from state 0 to state 2 (Pat-ID 4 and 5). For example, Pat-ID 5 might have died without response and Pat-ID 4 may not have achieved PR up to week 24. Any patient who neither reached state 1 nor state 2 (i.e., drop out before week 24 without response and without any of the events defined as state 2) would have been censored in state 0 (e.g., Pat-ID 7).

Considering the day of randomization as baseline (time=0), the probability that a randomized patient is in response at a time point *t* can be obtained by scrolling over the time axis and assessing the state at that time point for each patient. Thus, the PBR can be calculated as a function of time by applying time-to-event methodology similar to the well-known Kaplan–Meier plot. While the Kaplan–Meier plot estimates one right-censored time-to-event variable (e.g., overall survival or duration of response) from a fixed time point (e.g., time of randomization or time of first response) for all patients, the PBR function aggregates two time-to-event variables, namely time from randomization to first response and time from first response to subsequent failure [6], more details and an illustration using the hypothetical data from Fig. 2 are provided in supplementary material S2. In order to compare PBR between treatment arms, we calculated the difference of PBR curves (ruxolitinib minus BAT) with pointwise 95% confidence intervals. All calculations were performed using R-4.1.0.

## Results

**Fig. 3:**
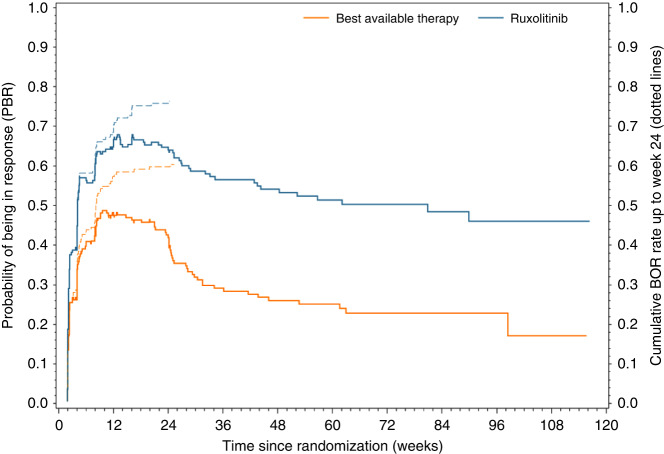
Probability of being in response (PBR) function of ruxolitinib versus BAT in REACH3. Comparison of PBR for ruxolitinib (blue) versus BAT (orange) in REACH3. Solid lines show PBR curves, dotted lines show cumulative number of patients with PR/CR up to week 24.

In REACH3, greater efficacy was observed on ruxolitinib versus BAT for most efficacy endpoints [2]. Summary results of the efficacy parameters are displayed in Table 1, more detailed information on the outcome of the pre-planned study endpoints is published in Zeiser et al. [2]. In addition, not reported previously, the median time to first response for all randomized patients (with non-responders censored) was 29 days (95% CI: 24 to 31 days) for ruxolitinib and 50 days (95% CI: 29 to 57 days) for BAT. A slightly earlier time to first response, a higher probability of being in response at all time points and a longer response duration for ruxolitinib than BAT is apparent from the PBR curves (Fig. 3). The higher clinical benefit of ruxolitinib is also visualized by a larger area under the curve.

**Table 1:** Summary of efficacy results in REACH3.

Endpoint	Population		Rux	BAT
ORR at Cycle 7 Day 1	All patients	*n* (%)	82 (49.7)	42 (25.6)
Failure-free survival (FFS)	All patients	Probability (95% CI) at		
		month 3 from randomization	83.6 (77.1, 88.5)	71.1 (63.3, 77.5)
		month 6 from randomization	74.9 (67.5, 80.9)	44.5 (36.5, 55.1)
		month 12 from randomization	64.0 (55.8, 71.1)	29.6 (22.3, 37.2)
Best overall response (BOR)	All patients	*n* (%)	126 (76.4)	99 (60.4)
Duration of response (DOR)	Responders	Probability (95% CI) at		
		month 6 from first response	76.6 (67.5, 83.2)	52.1 (41.8, 61.5)
		month 12 from first response	68.5 (58.9, 76.3)	40.3 (30.3, 50.5)
Time to first response (TTFR)		Days since randomization		
	(a) Responders	median (range)	20 (13, 170)	28 (13, 171)
	(b) All patients	median (95% CI)	29 (24, 31)	50 (29, 57)
Probability of being in response function (PBR)	All patients	Probability (95% CI) at		
		month 3 from randomization	67.9 (56.0, 79.8)	47.6 (38.0, 57.2)
		month 6 from randomization	62.0 (51.4, 72.7)	35.4 (28.2, 42.6)
		month 12 from randomization	53.3 (44.0, 62.6)	26.0 (20.6, 31.5)

All patients include all randomized subjects (*N* = 165 for ruxolitinib, *N* = 164 for BAT). Responders include all patients with BOR = CR or PR (*N* = 126 for ruxolitinib, *N* = 99 for BAT).

*BAT* best available therapy, *BOR* best overall response, *CI* confidence interval, *DOR* duration of response, *PBR* probability of being in response function, *TTFR* time to first response.

As per definition of PBR, the maximum of the curves is lower than the reported BOR (76.4% vs 60.4% for ruxolitinib and BAT, respectively) because PBR is estimated from the percentage of patients in response at the same point in time, whereas BOR is calculated from the best response, ignoring when this best response occurred and how long it was sustained. Calculating the cumulative number of patients with BOR = CR or PR up to week 24 reaches exactly the BOR rates at week 24 (dashed lines in Fig. 3). However, in contrast to PBR this naive cumulation ignores completely that patients may have lost their response before week 24 (e.g., Pat-ID 6 in Fig. 2) and is shown for comparative purpose only.

**Fig. 4:**
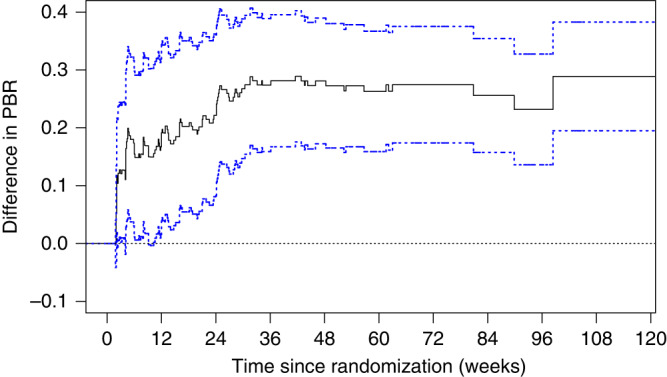
Difference of PBR with pointwise 95% confidence interval (difference refer to ruxolitinib − BAT). Difference in PBR (black line) with 95% CI (blue lines) between ruxolitinib and BAT.

The difference between PBR curves with confidence intervals, which provides a more formal comparison than the visual inspection of Fig. 3, clearly shows that superiority of ruxolitinib is achieved within very few weeks after randomization and is maintained over the entire study period (Fig. 4).

## Discussion

Although probability of being in response was considered as a useful method to assess response over time in the statistical literature during the last decades, very few applications can be found in clinical research. In one recent paper, Huang et al. used PBR (referred to as PBIR by Huang et al.) to compare different treatments for renal cell carcinoma [7].

In this post-hoc analysis, we applied PBR to the REACH3 study data to show the benefit of this method when assessing efficacy of cGvHD treatments. PBR provides easily interpretable curves presenting simultaneously the time from treatment start to first response and subsequent failure based on all randomized patients. Results obtained in REACH3 clearly illustrate the superiority of ruxolitinib versus BAT, further confirming the results reported in the original study publication [2]. PBR offers a visual comparison of efficacy over time between treatment arms based on all patients and the entire study period. In contrast, DOR estimates time from first response and visualizes duration of response for the subgroup of patients who responded to treatment only, which can result in a biased assessment of treatment benefit (for example, if a higher percentage of patients reaches the response state in the experimental than in the control arm).

The multistate model and the resulting estimated PBR function considered in this paper were defined in alignment with the definition of efficacy endpoints as pre-specified in the REACH3 study protocol. The design of this multistate model (PBR function), allowing transition from state 0 = *not in response* to state 1 = *in response* but NOT state 1 to state 0, as well as the definition of events for the end of response are based on exactly the same criteria as REACH3 efficacy endpoints.

In future work or for other studies, one could extend the model, or alternatively define events differently than done here for REACH3. In particular, as determined in the study protocol, patients were counted as responders only if the first response occurred up to week 24. However, first response to treatment may occur after week 24, i.e., patients who did not respond up to week 24 do not necessarily need to enter the absorbing state 2 at week 24. Furthermore, loss of response was aligned with the definition of DOR, i.e., once a patient enters state 1 = *in response*, the patient can either stay in that state until the analysis cut-off date or can lose the “in response” status by entering the absorbing state 2, thus ending the duration of response. However, in diseases such as cGvHD, it may also be meaningful to extend the model by allowing transitions from “in response” (state 1) back to “not in response” (state 0) before entering the absorbing state 2. If for instance a patient achieves an overall response of PR by improvement of cGvHD symptoms in several organs at week 8, but subsequently one organ worsened at week 12 (with response maintained in the other organs) and improved again at week 16 without having changed systematic cGvHD treatment, it would be reasonable to assign state 0 *= not in response*, state 1 = *in response*, state 0 *= not in response* and state 1 = *in response* at study start, week 8, week 12 and week 16, respectively. PBR could be applied to such model extensions.

One of the reasons why applications of PBR can hardly be found in the clinical literature may be the lack of statistical software to perform these analyses. Recently, Xiadong et al. provided the R-package PBIR which can be easily applied within the open-source software R [8]. Due to the special situation that cGvHD first response to treatment in REACH3 was counted up to week 24, we have generated our own R-codes and used PBIR R-package for validation purposes only (PBIR would have cut the curves at week 24).

It would also be useful to have a formal statistical test for comparing the treatments with respect to PBR. We do not elaborate on this topic here for two reasons. Firstly, the difference of PBR curves including pointwise 95% confidence intervals (Fig. 4) allows a good visual comparison between treatment arms, and provides sufficient evidence that the difference between the curves is statistically significant. Secondly, to our knowledge, a statistical test providing a direct generalization of the usual log-rank test is not yet available for the situation described in this paper; its development is subject of forthcoming work. Considering a parametric estimation of PBR curves using the exponential distribution, Ellis et al. proposed a statistical test by comparing the area under the PBR curves (referred to as expected duration of response, EDoR) between treatment arms [5]. Other alternatives (such as a log-rank test of time in response from entering the response state until leaving it for the absorbing state, potentially setting time in response to 0 for those patients who went from state 0 straight to state 2) are conceivable, but a thorough discussion of their properties, interpretational restrictions and precise relation with the PBR curve is beyond the scope of this paper.

As illustrated with the data of REACH3 we strongly believe that PBR can serve as a meaningful efficacy endpoint for the assessment of cGvHD treatments, in addition to ORR/BOR and failure-free survival (FFS) which are recommended as endpoints for clinical trials by the NIH clinical design working group [9]. Compared to these established endpoints PBR provides a more comprehensive summary of treatment efficacy because it integrates several aspects of the treatment benefit (time-to-response, duration of response) into a single measure. Whereas time is not taken into account for ORR/BOR, FFS describes the time to treatment failure only but does neither assess if patients respond to treatment nor the time to response. Further clinical input would be required to include PBR into an updated cGvHD response guideline, also to ensure that consistent criteria are applied across future clinical trials. For example, a clear definition of response duration and end of response would be required. The current guidelines postulate to ‘*document durability of response and to determine whether continued treatment is needed to maintain response*’ and state that ‘*Efforts to document the durability of response are strongly encouraged*’, but do not provide clear definitions of response durability [1, 9]. Finally, PBR represents a useful endpoint measure which could be applied for all diseases and indications, for which clinical benefit is assessed by response to treatment in the context of time, demonstrating further utility outside of the cGvHD treatment landscape.

### Supplementary information

Supplementary Material

## Data Availability

Individual data sharing to third parties will not be possible. Access to aggregated data might be granted following review. Such requests can be submitted to the corresponding author for consideration.
