# Supplementary material for: A novel approach to visualize clinical benefit of therapies for chronic graft versus host disease (cGvHD): the probability of being in response (PBR) applied to the REACH3 study
Source: Bone Marrow Transplant. 2023 Oct 28;59(1):12–6. doi: 10.1038/s41409-023-02128-8 (PMC10781633; doi:10.1038/s41409-023-02128-8)
Supplement: Supplementary file 1 — Supplementary Material [file 41409_2023_2128_MOESM1_ESM.docx]

**Supplementary material**

**Hollaender et al., A novel approach to visualize clinical benefit of therapies for chronic graft versus host disease (GvHD): the probability of being in response (PBR) applied to the REACH3 study.**

**S1: Efficacy endpoints in the REACH3 study**

| **Endpoint** | **Definition** | **Use in REACH3** | **Patients analyzed** |
| --- | --- | --- | --- |
| Overall response rate (ORR) at Cycle 7 Day 1 | Proportion of patients with complete response (CR) or partial response (PR) as per overall response assessed by the investigator at the Cycle 7 Day 1 visit (week 24), patient with change or addition of systemic cGvHD treatment before the assessment were counted as non-responder. | Primary endpoint | All randomized patients |
|  |  |  |  |
| Failure free survival (FFS) | Time from randomization to recurrence of the underlying disease, start of new systemic treatment for cGvHD, or death, whichever came first. For patients without any of these events, FFS is censored at the last contact date. | Key secondary endpoint | All randomized patients |
|  |  |  |  |
| Best overall response (BOR) | Proportion of patients who achieved overall response (CR or PR) at any time point up to and including Cycle 7 day 1 and before the change or addition of systemic cGvHD treatment. | Secondary endpoint | All randomized patients. Often used in publications to report response to cGvHD treatment |
|  |  |  |  |
| Duration of response (DOR) | Time from first response until cGvHD progression, death, or the start of new systemic cGvHD treatment for cGvHD, whichever came first. For patients without any of these events, DOR is censored at the last contact date. | Secondary endpoint | Analyzed for responders only (i.e., all subjects with BOR=CR or PR) |
|  |  |  |  |
| Time to first response (TTFR) | Time from date of randomization to the first documented overall response = CR or PR. (For Non-responders – if included - TTFR is censored at the earliest of the last assessment date or the Cycle 7 Day 1 visit). | Not done | Can be calculated for responders only, or for all randomized patients using time-to event analysis |
|  |  |  |  |
| Probability of being in response (PBR) | Combines TTFR, response rates and DOR to simultaneously estimate the time to first response and subsequent failure. For responders PBR aggregates TTFR and time from first response to failure, using the DOR events above to define the end of PBR. A responder with no observed event at the end of follow up is censored at the last contact date. Non-responders contribute only to the risk set of the PBR calculation (since they never reach the state of “in response”). They stop being a member of the risk set at the earliest of the following events: death, start of new systemic treatment for cGvHD, recurrence of the underlying disease, not having achieved a response up to week 24. | Not done | All randomized patients |
|  |  |  |  |

BOR, best overall response; CR, complete response; cGvHD, chronic graft-vs-host disease; DOR, duration of response; FFS, failure free survival; ORR, overall response rate; PBR, probability of being in response function PR, partial response; TTFR, time to first response.

**S2**

This supplement illustrates the aggregation of the two time to event endpoints (time to first response & time from first response to subsequent failure) to calculate PBR, using the toy data shown in figure 2.


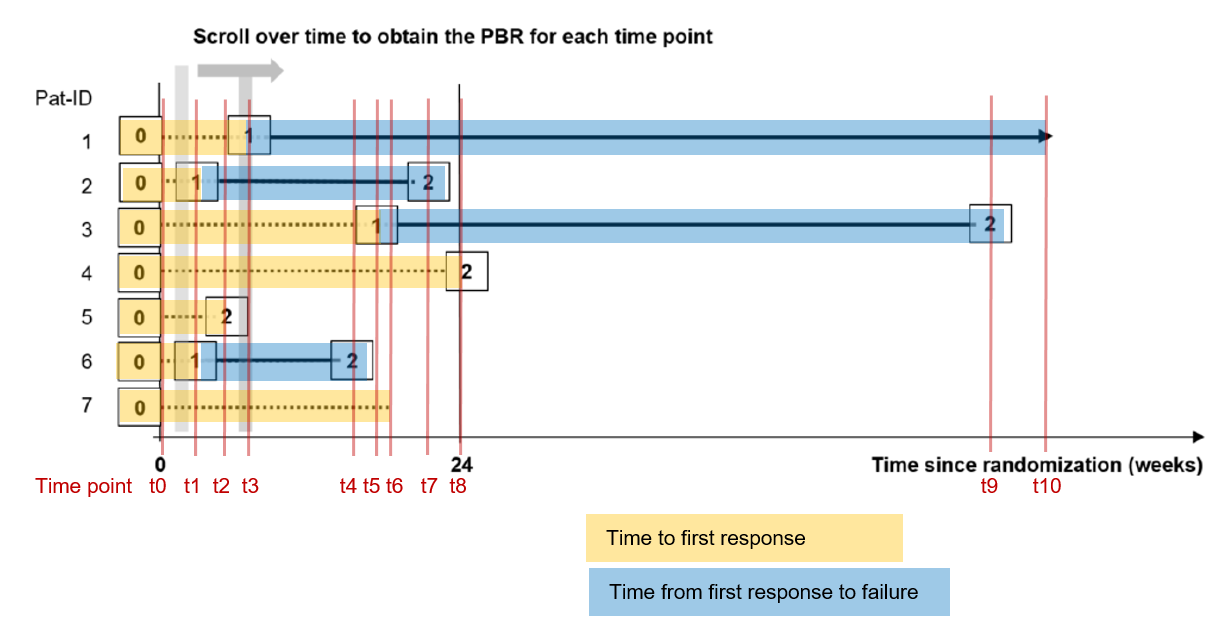


**PBR calculation**

At each time point t, PBR is obtained by adding i) the probability that a patient becomes a responder (enters state 1) at t and ii) the probability that a patient became a responder before t and did not lose the response up to time t.

Starting at time point t=t0=0 (all patients are in state 0, i.e. the probability of being in state 0, 1 and 2 is equal to 1, 0 and 0, respectively) PBR is calculated recursively for all subsequent time points with an event (i.e. at least one change of states 0 → 1, 0 → 2 and/or 1 → 2 is observed). Time points with a censored observation or with a 0 → 2 transition do not change the PBR value but impact the PBR curve at subsequent time points indirectly by reduction of the risk set.

More formally, we use the following notations

- P0 (t) = Probability of being in state 0 at time t
- **PBR (t) = P1 (t) = Probability of being in state 1 (=responder) at time t**
- d _i → j_ (t) = Number of patients who transition from state i to state j (i, j = 0,1,2 with i<j) at time t
- R0 (t) = No. of patients who can respond (“at risk” to become a responder), risk set decreases after each time point t with a transition from states 0 → 1, 0 → 2 and/or if a patient is censored in state 0
- R1 (t) = No. of patients who can lose the response (“at risk” to lose response), risk set increases at each time point t with a transition from states 0 → 1 and decreases after each time point t with a transition from states 1 → 2 or if a patient is censored in state 1
- For time points t with R0 (t) = 0, division by 0 is defined as x/0 =: 0

The table below shows data and results for the toy example.

The recursive calculation of P0 (t) and PBR (t) is illustrated for the first few time points thereafter.

| Time  point | d _i → j_ (t) = Number of patients who transition  from state | | | No. of patients censored | Total No. of patient left after the time point^+^ | R0 (t)  directly after the time point^+^ | P0 (t) | R1 (t)  directly after the time point^+^ | **PBR (t)**  **= P1 (t)** |
| --- | --- | --- | --- | --- | --- | --- | --- | --- | --- |
|  | 0 → 1 | 0 → 2 | 1 → 2 |  |  |  |  |  |  |
| t0 | 0 | 0 | 0 |  | 7 | 7 | 1 | 0 | 0 |
| t1 | 2 | 0 | 0 |  | 7 | 5 | 0.714 | 2 | 0.286 |
| t2 | 0 | 1 | 0 |  | 6 | 4 | 0.571 | 2 | 0.286 |
| t3 | 1 | 0 | 0 |  | 6 | 3 | 0.429 | 3 | 0.429 |
| t4 | 0 | 0 | 1 |  | 5 | 3 | 0.429 | 2 | 0.286 |
| t5 | 1 | 0 | 0 |  | 5 | 2 | 0.286 | 3 | 0.429 |
| t6 | 0 | 0 | 0 | 1^#^ | 4 | 1 | 0.286 | 3 | 0.429 |
| t7 | 0 | 0 | 1 |  | 3 | 1 | 0.286 | 2 | 0.286 |
| t8 | 0 | 1 | 0 |  | 2 | 0 | 0 | 2 | 0.286 |
| t9 | 0 | 0 | 1 |  | 1 | 0 | 0 | 1 | 0.143 |
| t10 | 0 | 0 | 0 | 1^##^ | 0 | 0 | 0 | 0 | 0.143 |

^+^ at the exact time point the risk set is equal to the previous time point, e.g. at t=t1 R0(t1)=7, directly thereafter (t>t1) R0(t) =5

^#^ Pat-ID 7 censored in state 0, ^##^ Pat-ID 1 censored in state 1

**Recursive calculation of P0 (t)**

P0 (t0) = 1

P0 (t1) = P0 (t0) x (1 - $\frac{d 0 \to1 \left( t1 \right)+ d 0 \to2 (t1)}{R0 \left( t1 \right)}$ ) = 1 x (1 - $\frac{2 + 0)}{7}$ ) = 1 x $\frac{5}{7}$ = 0.714

P0 (t2) = P0 (t1) x (1 - $\frac{d 0 \to1 \left( t2 \right)+ d 0 \to2 (t2)}{R0 \left( t2 \right)}$ ) = $\frac{5}{7}$x (1 - $\frac{0 + 1}{5}$ ) = $\frac{5}{7}$ x $\frac{4}{5}$= $\frac{4}{7}$ = 0.571

P0 (t3) = P0 (t2) x (1 - $\frac{d 0 \to1 \left( t3 \right)+ d 0 \to2 (t3)}{R0 \left( t3 \right)}$ ) = $\frac{4}{7}$ x (1 - $\frac{1 + 0}{4}$ ) = $\frac{4}{7}$ x $\frac{3}{4}$ = $\frac{3}{7}$ = 0.429

continue accordingly for time point t4 ff.

At each time point t, P0 (t) is obtained by multiplying the probability that a patient was in state 0 at the previous time point and the rate of patients who are still in state 0 at time t (i.e. did neither transition to state 1 nor 2). Note that P0 (t) is also used to calculate PBR (t) as described below.

**Recursive calculation of PBR (t) = P1 (t)**

P1 (t0) = 0

P1 (t1) = P0 (t0) x $\frac{d 0 \to1 \left( t1 \right)}{R0 \left( t1 \right)}$ ) + P1 (t0) x (1 - $\frac{d 1 \to2 \left( t1 \right)}{R1 \left( t1 \right)}$ ) = 1 x $\frac{2}{7}$ + 0 x (1 - $\frac{0}{0}$ ) = $\frac{2}{7}$ = 0.286

P1 (t2) = P0 (t1) x $\frac{d 0 \to1 \left( t2 \right)}{R0 \left( t2 \right)}$ ) + P1 (t1) x (1 - $\frac{d 1 \to2 \left( t2 \right)}{R1 \left( t2 \right)}$ ) = $\frac{5}{7}$ x $\frac{0}{5}$ + $\frac{2}{7}$ x (1 - $\frac{0}{2}$ ) = $\frac{2}{7}$ = 0.286

P1 (t3) = P0 (t2) x $\frac{d 0 \to1 \left( t3 \right)}{R0 \left( t3 \right)}$ ) + P1 (t2) x (1 - $\frac{d 1 \to2 \left( t3 \right)}{R1 \left( t3 \right)}$ ) = $\frac{4}{7}$ x $\frac{1}{4}$ + $\frac{2}{7}$ x (1 - $\frac{0}{2}$ ) = $\frac{1}{7}$ + $\frac{2}{7}$ = 0.429

continue accordingly for time point t4 ff.

At each time point t, PBR (t) is obtained by adding the probability that a patient was in state 0 at the previous time point and enters state 1 (=responder) at t and the probability that a patient was in state 1 at the previous time point and is still in state 1 at time t (i.e. did not transition to state 2).
